# Ionisable substances chromatography: A new approach for the determination of Ketoprofen, Etoricoxib, and Diclofenac sodium in pharmaceuticals using ion – pair HPLC

**DOI:** 10.1016/j.heliyon.2020.e04613

**Published:** 2020-08-04

**Authors:** Georgeos Andraws, Saleh Trefi

**Affiliations:** Pharmaceutical Quality and Pharmaceutical Chemistry Department, Faculty of Pharmacy, University of Aleppo, Syria

**Keywords:** HPLC, Ion pair liquid chromatography, Ketoprofen, Eetoricoxib, Diclofenac sodium, Chemistry, Analytical chemistry, Chromatography, Ion exchange, Pharmaceutical chemistry, Pharmaceutical science

## Abstract

An ion-pair HPLC method was developed and validated to analyze three of non-steroidal anti-inflammatory drugs (Ketoprofen, Etoricoxib, and Diclofenac sodium) in their pure and pharmaceuticals based on their ionisable characteristics. Cetyltrimethylammonium bromide (Cetrimide) was used as an ion pair reagent since it had not been used before for this purpose. Chromatographic analysis was accomplished using the C18 (250 × 4.6 mm, 5μm) column. Mobile phase consisted of a mixture of 50% Cetrimide$\;10^{-3}$ M and 50% acetonitrile to analyze Ketoprofen and Etoricoxib, whereas for Diclofenac sodium, mobile phase was a mixture of 30% Cetrimide$\;10^{-3}$ M and 70% acetonitrile. pH value was adjusted if necessary to 10 with ammonium hydroxide. The flow rate was 1mL/min and detection wavelengths were at 254 nm, 234 nm, and 254 nm for Ketoprofen, Etoricoxib, and Diclofenac sodium; respectively under ambient temperature. Retention times (${\text{R}}_{\text{t}}$) were 9.41, 7.34, and 6.66 for Ketoprofen, Etoricoxib, and Diclofenac sodium; respectively. The proposed method was evaluated for linearity, accuracy, precision, and specificity according to ICH guidelines. Ketoprofen, Etoricoxib, and Diclofenac sodium were detected in the following linear ranges: (0.031–0.500mg/mL), (0.007–0.110g/mL), and (0.016–0.250mg/mL); respectively with excellent mean recovery values (98.0–102.0%). RSD% was in an acceptable range (less than 2), proving the precision of the developed method. Specificity was proved in the presence of degradation products. Furthermore, a comparison between the results of this study and the reported HPLC methods indicated that this developed method was better in terms of simplicity, analysis time, and no use of buffers in the mobile phase. In conclusion, the developed method can successfully detect Ketoprofen, Etoricoxib, and Diclofenac sodium quantitatively and qualitatively in their dosage forms without any interference with excipients, making this method valuable, reliable, and practical to be applied in quality control laboratories.

## 1 Introduction

### 1.1 Ion-pair chromatography (IPC)

Ion-pair chromatography is a type of reverse-phase partition chromatography that is used for the separation of ionisable structured compounds. The eluent system used in IPC contains an ionic compound with a relatively large organic counter-ion for the analyzed ions, which can form a neutral ion-pair:

A^+^ (Ionsample) + B^−^ (Counter-ion) ↔ A^+^B^−^ (Ion-pair)

This ion-pair formed will behave as neutral species because it will be hydrophobic in character. As a result, this ion-pair will be attracted to the non-polar stationary phase [1].

There are three essential mechanisms proposed to describe the ion-pair performance: ion pair, ion exchange, and ion interaction.

A. Ion-pair mechanism:

This concept postulates the formation of a tightly bound ion-pair of zero charges. After adjusting the pH of the eluant, an ion-pair reagent (IPR) is added to the sample. This IPR contains a counter-ion (${\text{A}}^{-}$), which has the opposite charge to that of the compounds that would be determined and where subsequently an uncharged ion-pair will be formed.

HA (IPR) ↔ H^+^ + A^−^ + B^+^ ↔ {A−B}^0^ (Ion-pair)

B. Ion exchange mechanism:

This postulates that the column conducts as an ion exchange, whereas the lipophilic end of counter-ions effectively locates onto the bonded stationary phase.

C. Ion interaction mechanism:

This suggestion is based neither on ion-pair or ion exchange phenomena, though the lipophilic ions are adsorbed onto the surface, but are associated with a primary ion giving an electrical double layer. Then, an interaction will be occurred between the analyte and this double layer dynamically, by both electrostatic and van der Waal's type forces [2].

### 1.2 Ion-pair reagents (IPRs)

A non-polar surface (e.g. C8 or C18) is used as a stationary phase for reverse-phase ion-pair chromatography (RF-IP) and an ionic alkyl compound is added to the aqueous mobile phase as a modifier. An organic base (e.g. tetrabutyl ammonium phosphate) is added to the eluent for the separation of acids, whereas an organic acid (e.g. octane sulphonate) is used for the separation of bases.

There are a wide range of IPRs ranging from the anionic reagents to the cationic reagents. Some commonly encountered IPRs are presented in Table1 [3].

**Table 1:** Typical reagents employed in ion-pair chromatography.

Anionic counter-ion donors	Cationic counter-ion donors
• **Alkyl and aryl sulfonate:** Methanesulphonic acid (Na salt) Pentanesulphonic acid (Na salt) Hexanesulphonic acid (Na salt) Heptanesulphonic acid (Na salt) Octanesulphonic acid (Na salt) 2-Naphthalenesulphonic acid (Na salt) Dodecylsulphonic acid (Na salt) • **Alkyl sulfates:** Hexylsulfate Octylsulfate Decylsulfate Dodecylsulfate • **Inorganic:** Trifluoroacetate Trichloroacetate Phosphate	• **Quaternary amomium salts**$\;R_{4}N^{+}$**:** Tetramethylammonium hydroxide Tetraethylammonium hydroxide Tetrabutylammonium phosphate Hexadecyltrimethylammonium bromide Trihexylamine Triheptylamine Trioctylamine cetyltrimethylammonium bromide (cetrimide) • **Protonated ternary amines:** R_3_NH^+^

Cetrimide was selected as a cationic counter-ion donor for the analysis of different compounds under investigations (Non-Steroidal Anti Inflammatory Drugs; NSAIDs) as weak acids analytes.

The retention of analytes in IPC can be controlled in many ways: by modifying the solvent strength, varying the concentration of the IPR, varying the alkyl chain length of the counter-ion or by combining with ion suppression [4].

A lot of analytical methods were used by many researchers depending on ion-pair reagents to separate and analyze different chemical compounds; some of these methods are summarized and classified whether ion-pair reagents used are cationic or anionic:

Sodium phosphate buffer containing Octanesulfonic acid sodium salt (anionic IPR) and acetonitrile (86:14) was suggested as mobile phase by D. Bin Fan et al in 2002 to determine Zidovudine/Lamivudine/Nevirapine in human plasma using UV detection at 265 nm pH was adjusted to 3.2 with phosphoric acid [5]. In another study done by A. Zarghi et al., a rapid method was used to determine metformin in human plasma. Separation was performed on an analytical C18 (150 × 4.6 mm) column with UV detection at 235 nm. The mobile phase was 40% acetonitrile, 0.01 M Sodium dodecyl sulphate (SDS) (anionic IPR), 0.01 M sodium dihydrogen phosphate and distilled water to 100%. pH was adjusted to 5.1 at a flow rate of 1.5 mL/min [6]. In 2004, the same chromatographic conditions (column, mobile phase, and IPR) were used by A. Zarghi et al to determine Minoxidil in human plasma, where wavelength used was set at 281 nm pH was adjusted to 3.5 [7]. A method for the determination of gemcitabine beside its metabolite (dFdU) in plasma samples was introduced by R. Losa et al in 2005 basing on a C18 (300 × 3.9 mm, 10μm) column. The mobile phase consisted of Pentane-1-sulfonic acid (anionic IPR) and methanol (96:4) [8]. Risedronate in pharmaceutical preparations was analyzed in 2007 by D. Kyriakides and I. Panderi using a BDS C18 analytical column (250 × 4.6 mm, 5μm). The mobile phase composed of 0.005 M Tetrabutyl ammonium hydroxide (cationic IPR) and 0.005 M pyrophosphate sodium (pH = 7.0) mixed with acetonitrile in a ratio (78:22) [9]. The determination of azithromycin using Sodium heptane sulfonate (anionic IPR) was described by Z. Y. Yang et al in 2009. Mobile phase was ammonium dihydrogen phosphate (0.045 M, pH was adjusted to 3.0 by phosphoric acid): acetonitrile 47:15 (v/v) [10]. P. Jin et al presented the use of 25 mM ammonium dihydrogen phosphate (containing 0.01% Heptanesulfonic acid sodium salt as an anionic IPR) and acetonitrile (95:5, v/v) as the mobile phase to determine Condroitin sulfate sodium, Allantoin and pyridoxine hydrochloride in pharmaceutical eye drops [11]. In 2010, a method was employed to separate Desloratadine and related compounds in solid pharmaceutical formulations by J. Zheng and A. M. Rustum. The separation for Desloratadine was achieved by utilizing a C18 (150 mm × 4.6 mm I.D) column. The mobile phase (A) contained 3 mM Sodium dodecyl sulfate (SDS) (anionic IPR), 15 mM sodium citrate buffer (pH = 6.2) and 40 mM sodium sulfate, whereas the mobile phase (B) was acetonitrile [12]. Tributylamine was used as (cationic IPR) in 2010 by H. Kojima et al to separate lipopolysaccharide (LPS) related compounds using the reverse-phase ion-pairing chromatography [13]. In 2012, D. Zhang et al determined five components in compound a-ketoacid tablets. The separation was achieved with reverse-phase ion-paring chromatography (RPIP- HPLC). Tetra butyl ammonium hydroxide was used as (cationic IPR) [14]. Eberconazole Nitrate was estimated in bulk form and pharmaceutical dosage forms by M. Vamsi Krishna using Ion-Pair RP-HPLC Method. 75% of 10 mM potassium dihydrogen phosphate containing 10 mM tetra-butyl ammonium hydroxide (cationic IPR) and 25% of methanol were used as a mobile phase [15]. A high pH ion-pairing strategy was used in 2015 by J. R. Dentona for the chromatographic determination of 2-hydroxypyridine-1-oxide (HOPO) in pharmaceutically relevant materials using hydroxy benzotriazole (HOBt) as a coupling reagent (cationic IPR) [16]. Naproxen and Esomeprazole were estimated in pharmaceutical preparations depending on a novel ion-pair RP-IP method. The method was developed by R. Kayesh et al using an isocratic condition of mobile phase. The mobile phase comprised [tetrabutylammonium hydroxide (cationic IPR) and n-heptane sulfonic Acid-Na salt] as a buffer, acetonitrile, and methanol in a 60: 20: 20 v/v/v ratio [17]. Theophylline and salbutamol were validated using ion-pair liquid chromatography by S. L. Sophi. A mixture of acetic acid and methanol (60:40v/v) which contains 3.5mM sodium-1-octane sulphonate (anionic IPR) was used as mobile phase [18]. Ion-pair isocratic simultaneous determination of Fluoroquinolones in environmental samples was described by L. Hlabangan and S. Memeza using HPLC with UV detection. The mobile phase consisted of phosphate buffer (containing a mixture of potassium di-hydrogen phosphate, 1-Heptane sulphonic acid as an anionic IPR and sodium hydroxide) and 15–25 % acetonitrile [19]. An ion-pair RP-HPLC method was established in 2019 by M. A. Mahrousea and N. T. Lamie for the simultaneous determination of Metformin hydrochloride, Alogliptin benzoate and Repaglinide in tablets using acetonitrile: phosphate buffer (0.01 M, adjusted to pH 2.5 with o-phosphoric acid): sodium heptane sulfonate (anionic IPR) in water (60:20:20, v/v/v) as a mobile phase at flow rate 1 mL/min. The UV detection was carried out at 220 nm [20]. In the same year, isomers of impurities in phosphate diester oligonucleotides drugs were separated by S. G. Roussis et al. Alkyl amines of different lengths (cationic IPRs) were evaluated as reagents in ion-pair reverse-phase (IP-RP) method [21]. In 2016, S. Trefi assayed four psychotropic drugs Chlorpromazine, Clomipramine, Amitriptyline, and Nortriptylinein tablets by a single HPLC method. The chromatographic conditions were comprised of a classical C8-type column. The mobile phase contained 3g of Sodium lauryl sulfate (anionic IPR) in a mixture of 400 mL of deionized water and 600 mL of acetonitrile. Then, 0.5 g of ammonium nitrate was added and an apparent pH of 3.0 was adjusted with glacial acetic acid [22]. A recent study was achieved in 2019 by L. Hammash, Y. Bitar and S. Trefi to separate pioglitazone hydrochloride and Sitagliptin phosphate in pure and tablet forms. The analysis of Pioglitazone hydrochloride and Sitgliptin phosphate was performed depending on (Method A); the chromatographic conditions were comprised of a C18 (250 × 4.6 mm, 5μm) column. The mobile phase was Sodium hexane sulfonic acid$\text{ ​}10^{-2}$M (anionic IPR) in a mixture of 500 mL of deionized water and 500 mL of acetonitrile. pH was adjusted to 2.5 with phosphoric acid. (Method B) was an additional method for pioglitazone hydrochloride analysis. The mobile phase composed of Cetrimide$10^{-3}$ M (cationic IPR) in a mixture of 400 mL of deionized water and 600 mL of acetonitrile [23]. In the same year, Sacubitril-Valsartan Combination of tablets was separated by S. Trefi, Y. Bitar, and V. Gilard. Mobile phase consisted of a mixture of 45% cetrimide$10^{-3}$M as (cationic IPR) and 55% acetonitrile [24]. Atorvastatin and ezetimibe were analyzed as a combination in tablets by S. Trefi. C18-type stationary phase with UV detection was used. The mobile phase consisted of a 35% of Cetrimide$10^{-3}$ M (cationic IPR) and 65% acetonitrile. The pH value of the mobile phase was adjusted if necessary by ammonia solution at 10 [25]. A novel two-dimensional liquid Chromatography-Mass spectrometry method was suggested by Z. Long et al for the direct identification of drug impurity from HPLC eluent. The mobile phase contained sodium 1-octanesulfonate (anionic IPR) and non-volatile buffer [26].

To best of our knowledge, cetrimide had been used as a reagent by our group to analyze pioglitazone hydrochloride, Sacubitril, Valsartan, Atorvastatin, and ezetimibe so it was selected and used for the first time in this study, as a unique cationic ion-pair reagent for the analysis of three different types of compounds belonging to NSAIDs using a single ion-pair HPLC method.

### 1.3 Ionisable substances

In this study, three of NSAIDs (Ketoprofen, Etoricoxib, and Diclofenac sodium) were chosen as ionisable substances for the formation of ion-pair complex with an IPR.

NSAIDs including Ketoprofen, Etoricoxib, and Diclofenac sodium are prescribed on a wide scale for the treatment of rheumatic arthritis and other degenerative inflammatory joint diseases. Although NSAIDs are very effective in relieving mild to moderate pains and inflammation, their use is often associated with many undesirable side effects, including bleeding, GI irritation, platelet dysfunction, bronchospasm, and kidney damage. Therapeutic effects of these drugs are considered to be mainly related to their inhibitory action on the COX-2 isozyme (COX-2: the inducible cyclooxygenase isozyme), whereas the undesirable side effects of the conventional NSAIDs are a result of inhibition of the COX-1 isozyme (COX-1: the constitutive cyclooxygenase isozyme). This hypothesis has stimulated extensive drug development and hasty market introductions of many selective COX-2 inhibitors, or Coxibs drugs [27].

Modulating painfulness coefficient, attenuating inflammation, and reducing pyrexia are maintained as therapeutic effects of the conventional NSAIDs by inhibiting both of cyclooxygenase isoforms (COX-1 and COX-2) which is the rate-limiting enzyme responsible for the biosynthesis of the pro inflammatory prostaglandins (PGs) such as the PGD2, PGE2, PGF2, and PGI2. GI bleeding, ulcerations or renal impairments is produced as undesirable side effects by blocking the same cyclooxygenases responsible for synthesizing PGs that modulate platelet activity (TXA2 and PGI2), gastric acid secretion, cytoprotection (PGE2 and PGI2), and renal blood flow (PGE2) [28].

**Figure 1:**
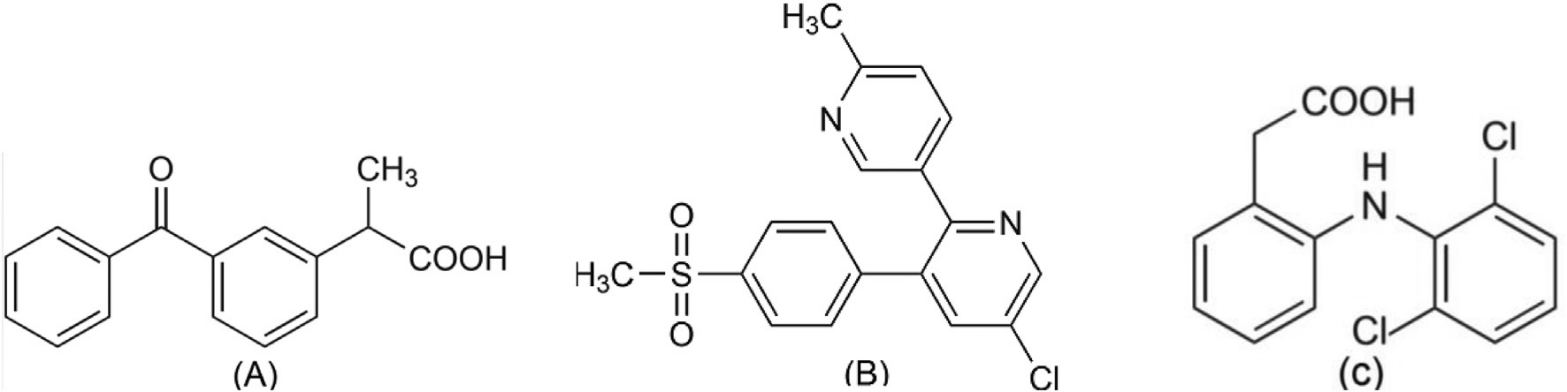
Chemical structures of Ketoprofen, Etoricoxib, and Diclofenac.

Ketoprofen (Figure 1A), is one of Aryl- and Heteroarylpropanoic acids class of NSAIDs. Even though ketoprofen has been approved for OTC use, it has GI side effects; therefore, its use should be closely monitored especially in patients with GI or renal problems. Etoricoxib (Figure 1B), which is one of the Coxibs that are a part of NSAIDs, is based on a hypothesis that says blocking the inducible COX-2 isozyme led to retain all of the therapeutic effects but none of the side effects of the conventional NSAIDs. Diclofenac sodium (Figure 1C), is one of the N-arylanthranilic acids (fenamates) class of NSAIDs. Unlike the other NSAIDs, Diclofenac appears to be more hepatotoxic and, in rare cases, can cause severe liver damage [29].

The recommended analytical method to analyze Ketoprofen by the Europian Pharmacopeia is HPLC. The analysis was performed using C18 (150 × 4.6 mm, 5μm) column. The mobile phase consisted of a mixture of Phosphate buffer: acetonitrile: water (2:43:55v/v/v). The detection wavelength was at 233 nm. The flow rate was 1 mL/min. The volume of injection was 20 μL and the retention times (${\text{R}}_{\text{t}}$) was 7min [30]. There were also many chromatographic methods for the determination of Ketoprofen in pharmaceuticals or biological Samples. These methods included HPLC estimation [31], UV spectrophotometry [32, 33], and capillary chromatography [34]. There were also many chromatographic methods utilized to assay of Etoricoxib depending on RP-HPLC [35]. Capillary zone electrophoresis [36] was used in another study for the comparative determination of Etoricoxib in pharmaceuticals. LC-tandem MS/MS method was used for the determination of Etoricoxib in human plasma and pharmaceuticals [37]. Diclofenac salt was determined in bulk and capsule dosage form by UV spectrophotometric method [38]. Many methods were developed for the determination of Diclofenac in human plasma using GCMS [39, 40]. Spectrophotometric analysis was used in a lot of methods for the determination of Diclofenac in pure form and pharmaceutical preparations [41, 42].

## 2 Experimental

### 2.1 Chemicals and reagents

The working standards of Ketoprofen and Etoricoxib were supplied by Ibn-Alhaytham Industries, Aleppo-Syria, whereas the working standard of Diclofenac sodium was supplied by from Razi Industries, Aleppo-Syria. Pharmaceutical samples were: Toricox® tablets (60mg Etoricoxib, produced by Unipharma Industries, Damascus-Syria), Etoxia® tablets (90mg Etoricoxib, produced by Razi Industries, Aleppo-Syria), Profenid® capsules (50mg Ketoprofen, produced by Oubri Industries, Aleppo-Syria), Diclorism® tablets (50mg Diclofenac sodium, produced by Shifa Industries, Aleppo-Syria), and Diclofenac Avenzor® ampoules (75mg Diclofenac sodium, produced by Avenzor, Damascus-Syria). All samples were stored in dark at ambient temperature and humidity. They were all analyzed within the expiry dates. All the other used reagents were of HPLC grade: acetonitrile (Biosolve, France), Cetrimide (Tnn, China), methanol (Biosolve, France), deionized water for HPLC, and syringe filters 0.45μm millipore membrane.

### 2.2 Equipment and software

In this study, the chromatographic system consisted of an Agilent (1260 infinity, Germany) with a vacuum degasser and UV detector. The separation was carried out on C18 (250 × 4.6 mm, 5μm) column. pH of mobile phase was checked using a pH meter from Crison (Madrid, Spain). Also, the ultrasonic processor (power sonic, model 405, Korea) and analytical balance ±0.1mg (Sartorius, model 2215, Germany) were used for the preparation of samples. Furthermore, Nylon 66 membranes (0.45μm pore size, 47.0 mm diameter) were obtained from SUPELCO, Bellefonte, USA. All glassware was cleaned with distilled water and dried in hot air oven whenever required. The solvents were filtered and degassed before use.

### 2.3 Solutions preparation

#### 2.3.1 Mobile phase solution

An amount of (0.336 g) of Cetrimide was dissolved in 1000 mL purified water in a 1000 mL volumetric flask. Then, the pH of this solution was adjusted if necessary to 10 with ammonium hydroxide and sonicated for 15 min. The final solution was then filtered with filter paper.

#### 2.3.2 Reference solutions

To prepare the starting standard solution of Ketoprofen, 100 mg of the working standard was transferred into a 100 mL volumetric flask and dissolved in 80 mL of methanol (solvent) and was sonicated for 10 min. Then it was diluted to the final volume to obtain the following starting standard solution for Ketoprofen (C = 1 mg/mL).

To prepare the starting standard solution of Etoricoxib, 47mg of the working standard was transferred into a 100 mL volumetric flask and dissolved in 80 mL of methanol (solvent) and was sonicated for 10 min. Then it was diluted to the final volume to obtain the following starting standard solution for Etoricoxib (C = 0.47 mg/mL).

To prepare the starting standard solution of Diclofenac sodium, 50mg of the working standards of Diclofenac sodium was transferred into a 100 mL volumetric flask and dissolved in 80 mL of distilled water (solvent) and was sonicated for 10 min. Then it was diluted to the final volume to obtain the following starting standard solution for Etoricoxib (C = 0.50 mg/mL). These starting standards solutions were used for the preparation of all diluted linearity solutions.

#### 2.3.3 Samples solutions

##### 2.3.3.1 Samples solutions of the assay study

Profenid®: an average powder content of 20 capsules was determined. Then, an amount of this powder, which was equivalent to the labeled content of one capsule, was transferred into a 50 mL volumetric flask and was dissolved using methanol. The content was dispersed under magnetic stirring for 20 min and was sonicated for 10 min until the active pharmaceutical ingredient was well dissolved. Then, the volume was diluted with methanol to a final concentration of (C = 0.2 mg/mL) within the linearity range.

Toricox®: 20 tablets of Toricox® samples were crushed and powdered. After that, an amount of these powders, which it was equivalent to the labeled content of one tablet, was transferred into a 25 mL volumetric flask and was dissolved using methanol. The content was dispersed under magnetic stirring for 20 min and was sonicated for 10 min until the active pharmaceutical ingredient was well dissolved. Then, the volume was diluted with methanol to get a final concentration in the range of linearity (C = 0.024 mg/mL).

Etoxia®: 20 tablets of Toricox® samples were crushed and powdered. The same procedures were conducted to get a final concentration of (C = 0.024 mg/mL) in the range of linearity.

Diclorism®: 20 tablets of Diclorism® samples were crushed and powdered. After that, an amount of these powders, which was equivalent to the labeled content of one tablet, was transferred into a 25 mL volumetric flask and was dissolved using water. The content was dispersed under magnetic stirring for 20 min and was sonicated for 10 min until the active pharmaceutical ingredient was well dissolved. Then, the volume was diluted with water to a final concentration of (C = 0.05 mg/mL) within the linearity range.

Diclofenac Avenzor®: the content of 5 ampoules of Diclofenac Avenzor® was emptied. Then, 3 mL (equivalent to one ampoule) was transferred into a 100 mL volumetric flask and was dissolved using water. The volume was adjusted with water to reaching a final concentration in the range of linearity (C = 0.075 mg/mL).

##### 2.3.3.2 Samples solutions of the ion-pair HPLC performance study

Profenid®: an average powder content of 3 capsules was determined. Then, an amount of this powder, which was equivalent to the labeled content of one capsule (50mg), was transferred into a 100 mL volumetric flask and was dissolved using methanol. The content was dispersed under magnetic stirring for 20 min and was sonicated for 10 min. Then, the volume was diluted with methanol to get a final concentration of (C = 0.5 mg/mL).

Etoxia®: 3 tablets were crushed and powdered. After that, an amount of these powders, equivalent to the labeled content of one tablet (90mg) was transferred into a 100 mL volumetric flask and was dissolved using methanol. The content was dispersed under magnetic stirring for 20 min and was sonicated for 10 min. Then, the volume was diluted with methanol to get a final concentration of (C = 0.9 mg/mL).

Diclofenac Avenzor®: the content of 3ampoules was emptied. Then, 3mL was transferred into a 100 mL volumetric flask and was dissolved using water. The volume was adjusted with water to reaching a final concentration in the range of linearity (C = 0.75 mg/mL).

All previous solutions (C = 0.5 mg/mL, C = 0.9 mg/mL, C = 0.75 mg/mL) were considered as starting solutions and were used to prepare all diluted solutions for repeatability and reproducibility studies for Ketoprofen, Etoricoxib, and Diclofenac sodium; respectively.

## 3 Results and discussion

### 3.1 Method development and optimization of chromatographic conditions

#### 3.1.1 Selection of column and wavelength detection

The chromatographic analysis was performed using a suitable column for the separation of studied compounds. Therefore, C18 (octadecylsilane) reverse-phase column was chosen to analyze Ketoprofen, Etoricoxib, and Diclofenac sodium. UV detection window was set at the wavelength of maximum UV signals produced by the studied compound. Both Ketoprofen and Diclofenac sodium showed maximum absorbance at 254nm, whereas Etoricoxib showed maximum absorbance at 234 nm.

#### 3.1.2 Mobile phase composition

**Figure 2:**
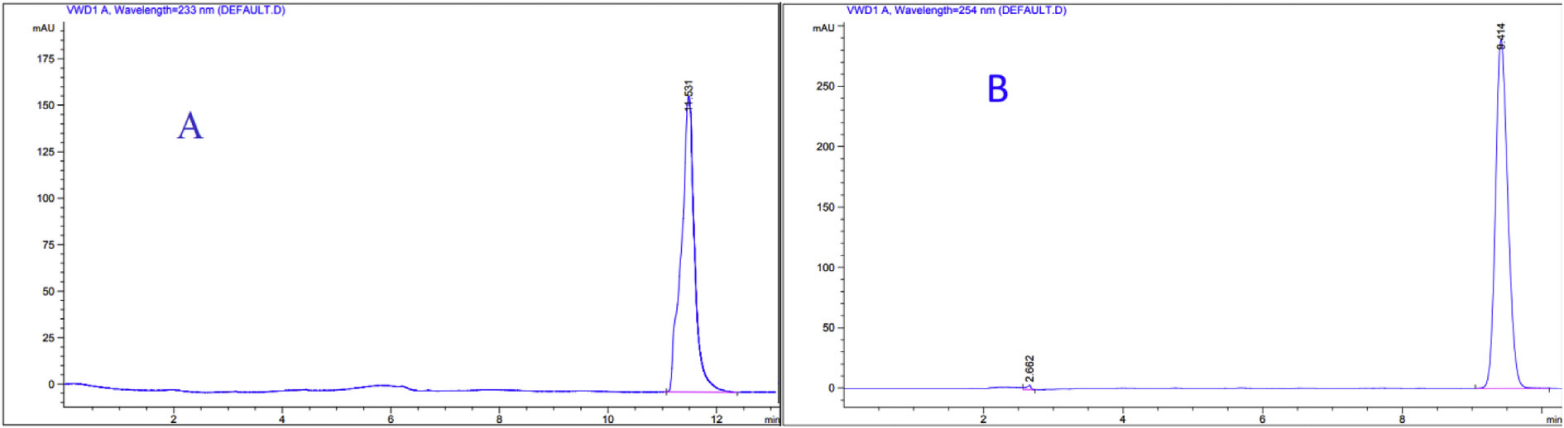
Comparison between tow chromatograms for the analysis of Ketoprofen using: (A) acetonitrile: water (40:60), (B) acetonitrile: water (50:50) (the optimum rate).

**Figure 3:**
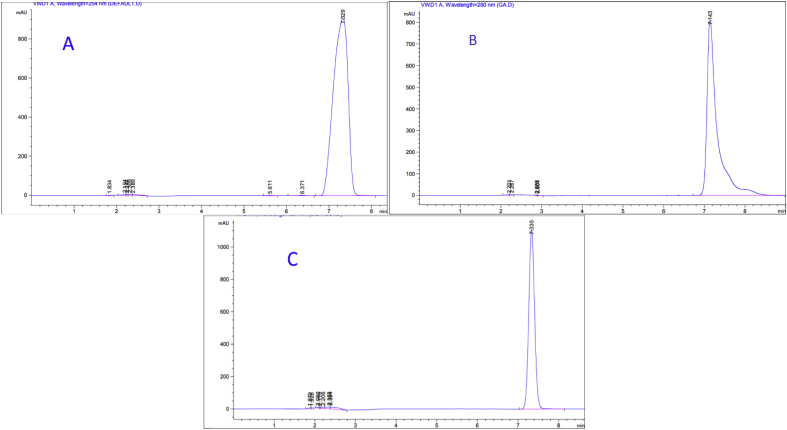
Comparison among different chromatograms for the analysis of Etoricoxib using: (A) acetonitrile: water (40:60), (B) acetonitrile: water (45:65), (C) acetonitrile: water (50:50) (the optimum rate).

**Figure 4:**
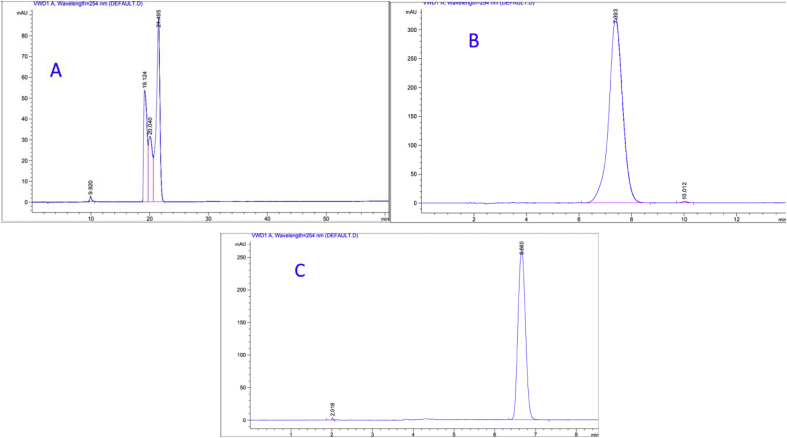
Comparison among deferent chromatograms for the analysis of Diclofenac sodium using: (A) acetonitrile: water (50:50), (B) acetonitrile: water (60:40), (C) acetonitrile: water (70:30) (the optimum rate).

There are different mobile phases such as water-methanol or water-acetonitrile as they are the solvents of choice for NSAIDs. Different rates of water and acetonitrile were tested and the optimum rate was chosen after carrying out various optimization experiments to get a symmetry peak shape with high resolution, best separation efficiency, and less retention time. To analyze Ketoprofen, a rate of (40:60) acetonitrile: water was tested. A wide, asymmetry peak shape was observed with large retention time (Figure 2A). A sharp and symmetric peak with an appropriate retention time was observed at a higher rate of acetonitrile (Figure 2B). Regarding Etoricoxib, a rate of (40:60) acetonitrile: water was tested. A wide peak was showed (Figure 3A). Also, an asymmetric peak was observed at a rate of (45:55) acetonitrile: water (Figure 3B). Therefore, best separation efficiency, sharp and symmetric peak shape was observed with a rate of (50:50) acetonitrile: water (Figure 3C). As for Diclofenac sodium a rate of (50:50) acetonitrile: water was tested. Superimpose peaks shape was noticed with long retention time (Figure 4A). The Shorter retention time was observed at a higher rate of acetonitrile (Figure 4B). Finally, a rate of (70:30) acetonitrile: water was selected to get a sharp and symmetric peak with an appropriate retention time (Figure 4C). Cetrimide concentration in the mobile phase significantly affects retention time of Ketoprofen, Etoricoxib, and Diclofenac sodium. The longer retention time was observed at higher concentrations. Therefore, the lowest possible concentration of Cetrimide (0.001%) was selected in final experimental conditions. Results obtained in these experiments are similar to other previous findings. For example, Alessia et al., 2006 [43] developed a method for the determination of Ketoprofen using acetonitrile and water (0.1% acetic acid) as a mobile phase in the same ratio of our approach (50:50). Manish et al., 2011 [44] used acetonitrile and water (0.05M KH2PO4 buffer) as a mobile phase in the ratio (50:50) for the determination of Etoricoxib in bulk and tablet dosage forms. In 2013, a mobile phase consisted of (phosphate buffer and acetonitrile) in the ratio (70:30) was used by Prinesh [45] for the simultaneous determination of Diclofenac. In their experiments, this mobile phase composition also generated similar well-resolved peaks as obtained in this investigation.

### 3.2 HPLC analysis

**Figure 5:**
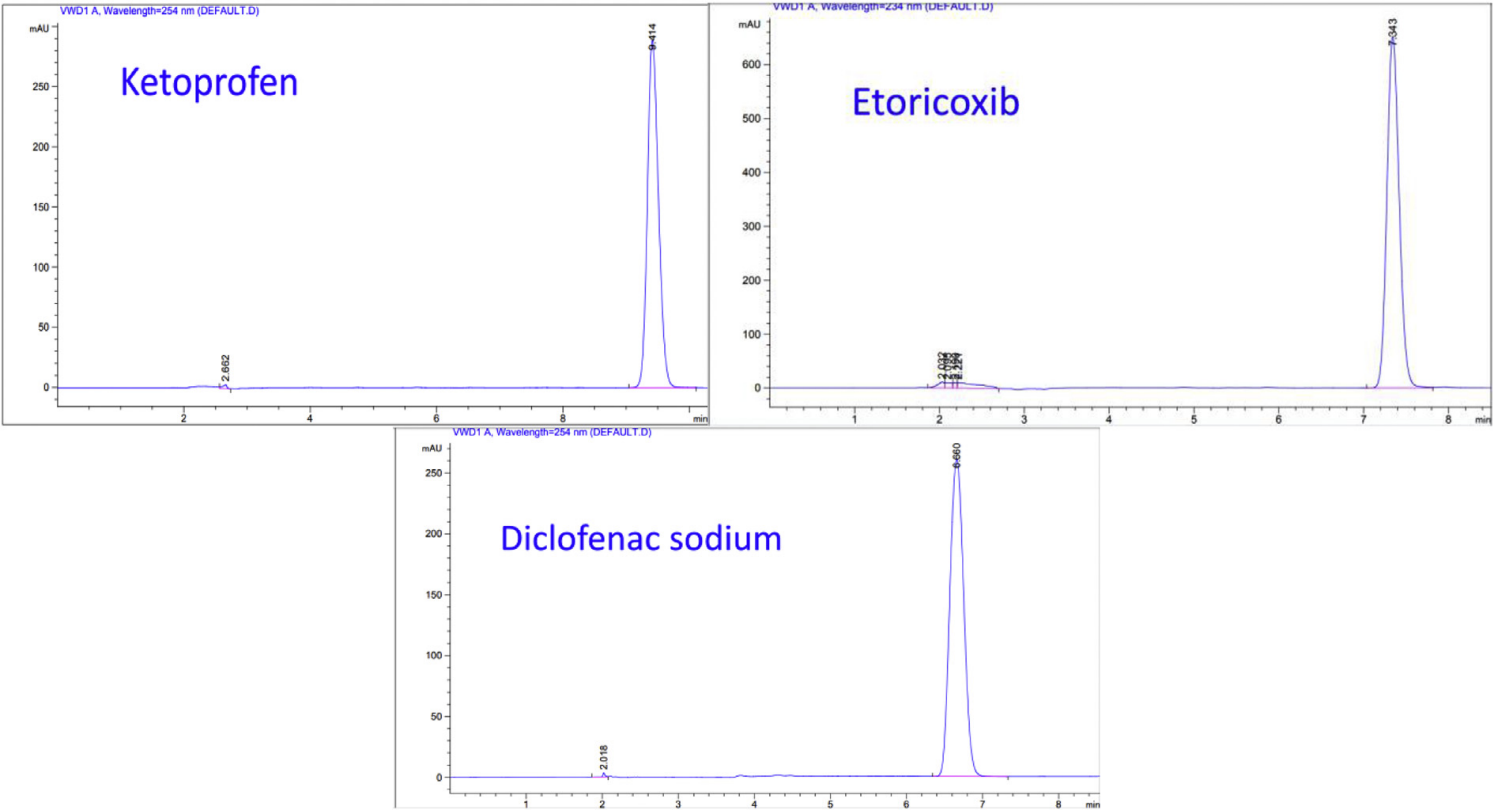
Chromatograms of standard solutions of (0.50 mg/mL), (0.11 mg/mL), and (0.25 mg/mL) for Ketoprofen, Etoricoxib, and Diclofenac sodium; respectively after the application of the optimal conditions.

In this study, the chromatographic analysis was accomplished using C18 reverse-phase (250 × 4.6 mm, 5μm) column. The optimal conditions to analyze Ketoprofen were: a mixture of 50% Cetrimide$\;10^{-3}$ M and 50% acetonitrile as a mobile phase, the pH was adjusted if necessary to 10 with ammonium hydroxide. The flow rate was 1 mL/min; the detection wavelength was 254 nm. The run time was set to 10 min and the column temperature was 25C. The reference solution was injected under the previous chromatographic conditions and the retention time (${\text{R}}_{\text{t}}$) was 9.41 for Ketoprofen (Figure 5). Regarding Etoricoxib, the optimal conditions were: a mixture of 50% Cetrimide$\;10^{-3}$ M and 50% acetonitrile as a mobile phase, the pH was adjusted if necessary to 10 with ammonium hydroxide. The flow rate was 1 mL/min; the detection wavelength was 234 nm. The run time was set to 10 min and the column temperature was 25C. The reference solution was injected under the previous chromatographic conditions and the retention time (${\text{R}}_{\text{t}}$) was 7.34 for Etoricoxib (Figure 5). As for Diclofenac sodium, the optimal conditions were: a mixture of 30% Cetrimide$\;10^{-3}$ M and 70% acetonitrile as a mobile phase, the pH was adjusted if necessary to 10 with ammonium hydroxide. The flow rate was 1 mL/min; the detection wavelength was 254 nm. The run time was set to 10 min and the column temperature was 25. The reference solution was injected under the previous chromatographic conditions and the retention time (${\text{R}}_{\text{t}}$) was 6.66 for Diclofenac sodium (Figure 5). The optimal conditions to analyze Ketoprofen, Etoricoxib, and Diclofenac sodium were summarized and listed in Table 2.

**Table 2:** The optimal conditions to analyze Ketoprofen, Etoricoxib, and Diclofenac sodium.

	Ketoprofen	Etoricoxib	Diclofenac sodium
Column (mm∗ μm)	(250 × 4.6)	(250 × 4.6)	(250 × 4.6)
Column temperature (C)	25	25	25
Mobile phase	Cetrimide$\;10^{-3}$ M: acetonitrile (50:50)	Cetrimide$\;10^{-3}$ M: acetonitrile (50:50)	Cetrimide$\;10^{-3}$ M: acetonitrile (30:70)
pH	10	10	10
Detection wavelength (nm)	254	234	254
Flow rate (mL/min)	1	1	1
run time (min)	10	10	10

### 3.3 Method validation

Validation of the developed method was carried out with respect to the following parameters: linearity, accuracy, precision, and specificity, depending on the international conference on harmonization (ICH) recommendations [46].

#### 3.3.1 Linearity and range

The linearity of the proposed method was evaluated by the analysis of working standard solutions of Ketoprofen, Etoricoxib, and Diclofenac sodium at five different concentrations within the working range. Each concentration was injected three times. This process was repeated three different times within three weeks.

**Figure 6:**
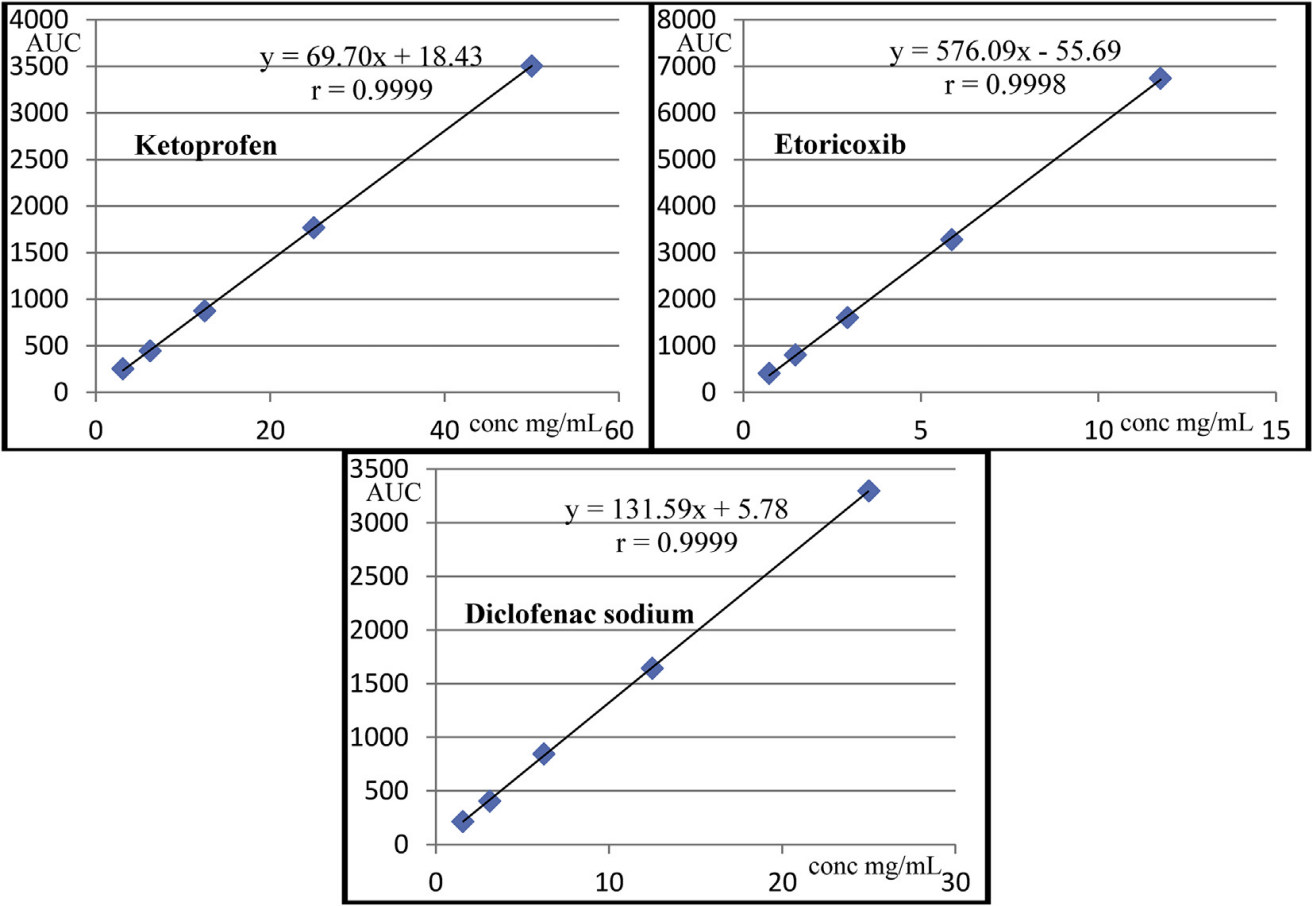
Linearity of Ketoprofen, Etoricoxib, and Diclofenac sodium.

The representative line of the relation between the areas under the curve (AUC) against the corresponding concentrations (C) for each drug was constructed. Then, the correlation coefficient (r) was calculated to evaluate the linearity of the method. Ideally, a calibration curve should be linear with an (r) value of 0.999 [47]. The equation of the calibration curve based on the peak response was y = 69.70x +18.43 with (r) of 0.9999 for Ketoprofen, y = 576.09x -55.69 with (r) of 0.9998 for Etoricoxib, and y = 131.59x +5.78 with (r) of 0.9999 for Diclofenac sodium. Results showing excellent correlations within the tested concentrations ranges and that suggest the linearity of the proposed method. The calibration curves were linear over the ranges (0.031–0.500 mg/mL) for Ketoprofen, (0.007–0.110 ​mg/mL) for Etoricoxib, and (0.016–0.250 mg/mL) for Diclofenac sodium. Regression lines of Ketoprofen, Etoricoxib, and Diclofenac sodium with the correlation coefficients (r) were shown in (Figure 6). Correlation coefficients, regression equations, and ranges were listed in Table 3.

**Table 3:** Correlation coefficients, Regression equations, and ranges of Ketoprofen, Etoricoxib, and Diclofenac sodium.

	Ketoprofen	Etoricoxib	Diclofenac sodium
Correlation coefficients (r)^a^	0.9999	0.9998	0.9999
Regression equations^a^	y = 69.70x +18.43	y = 576.09x -55.69	y = 131.59x +5.78
Ranges (mg/mL)	0.031–0.500	0.007–0.110	0.016–0.250

a n = 3: five concentrations over the working range.

#### 3.3.2 Accuracy

Accuracy of an analytical procedure expresses the closeness of agreement between the value which is accepted either as a conventional true value or an accepted reference value and the value found. For the quantitative approaches, at least nine determinations across the specified range should be obtained [46].

In this approach, three different levels of the following standard solution concentrations: (0.250, 0.125, and 0.062 mg/mL), (0.058, 0.029, and 0.014mg/mL), and (0.125, 0.062, and 0.031mg/mL) were used to study the accuracy of Ketoprofen, Etoricoxib, and Diclofenac sodium; respectively. The three levels of each drug were injected three times. This process was repeated three different times within three weeks. Recovery was determined by comparing the obtained concentration with the nominal concentration. Recovery of Ketoprofen ranged between (98.08% and 100.33%), whereas for Etoricoxib recovery was (98.16%–101.72%), and the recovery of Diclofenac sodium ranged from (99.07%–101.93%). The RSDs% values were 1.25, 1.98, and 2.43 for Ketoprofen, Etoricoxib, and Diclofenac sodium; respectively. The RSDs% for all tested drugs did not exceed (2.5%). The mean recovery values for all tested drugs were among the accepted range of accuracy (98–102%), therefore, the recovery of the proposed method was accepted and the developed method was accurate and applicable to the determination of Ketoprofen, Etoricoxib, and Diclofenac sodium. Table 4 shows in detail all accepted ranges, recovery and RSD% values for all studied drugs.

**Table 4:** Accuracy of the developed HPLC method.

Level mg/mL	Ketoprofen			Etoricoxib			Diclofenac sodium		
	(0.250) level_1_	(0.125) level_2_	(0.062) level_3_	(0.058) level_1_	(0.029) level_2_	(0.014) level_3_	(0.125) level_1_	(0.062) level_2_	(0.031) level_3_
Concentrations	0.250	0.122	0.061	0.057	0.028	0.014	0.124	0.063	0.030
	0.249	0.122	0.061	0.056	0.028	0.015	0.124	0.063	0.030
	0.255	0.123	0.061	0.057	0.028	0.014	0.124	0.063	0.030
	0.245	0.123	0.061	0.059	0.028	0.014	0.124	0.063	0.030
	0.251	0.122	0.060	0.059	0.028	0.014	0.124	0.063	0.030
	0.252	0.122	0.061	0.058	0.028	0.014	0.124	0.063	0.030
	0.249	0.123	0.060	0.056	0.028	0.014	0.124	0.063	0.030
	0.251	0.122	0.061	0.056	0.028	0.014	0.124	0.063	0.030
	0.250	0.123	0.061	0.057	0.028	0.014	0.124	0.063	0.030
Mean^a^	0.250	0.122	0.061	0.057	0.028	0.014	0.124	0.063	0.030
Theoretical concentration	0.250	0.125	0.062	0.058	0.029	0.014	0.125	0.062	0.031
Recovery %	100.33	98.29	98.08	98. 47	98.16	101.72	99.47	101.93	97.07
Mean recovery % ±SD	98.90 ± 1.24			99.45 ± 1.97			99.49 ± 2.42		
RSD%	1.25			1.98			2.43		

a n = 9.

#### 3.3.3 Precision

There are various levels of precision: repeatability, intermediate precision, reproducibility [46].

Repeatability and intermediate precision were confirmed by the determination of the following concentrations: 0.125 mg/mL, 0.029 mg/mL, and 0.062 mg/mL for Ketoprofen, Etoricoxib, and Diclofenac sodium; respectively and were expressed as RSD%. Ideally, the RSD% value should be less than 2% [47]. The measurement of 9 replicates of a previously fixed concentration was repeated during a period of three days including 3 replicates per day and their corresponding responses were recorded (short-term precision). The results were shown in Table 5.

**Table 5:** Results of short-term precision of the developed HPLC method.

N	AUC (mAU)									Mean^a^ ±SD	RSD%
	1	2	3	4	5	6	7	8	9		
Ketoprofen (0.125 mg/mL)	871.1	870.1	877.4	879.6	873.2	873.8	879.8	871.7	876.7	874.8 ± 3.6	0.41
Etoricoxib (0.029 mg/mL)	1606.4	1607.08	1606.5	1608.5	1606.5	1605.0	1601.1	1605.4	1602.5	1605.4 ± 2.3	0.14
Diclofenac sodium (0.062 mg/mL)	844.8	844.3	845.0	843.9	843.7	842.2	844.7	844.0	844.0	844.1 ± 0.8	0.09

a n = 9.

The RSDs of the results were 0.41%, 0.14%, and 0.09% for Ketoprofen, Etoricoxib, and Diclofenac sodium; respectively. The RSDs were less than 2.0% suggesting that the results were precise for the study.

#### 3.3.4 Specificity

Specificity is the ability to assess unequivocally the analyte in the presence of other components which may coexist like impurities, degradants, matrix, etc [46, 48].

**Figure 7:**
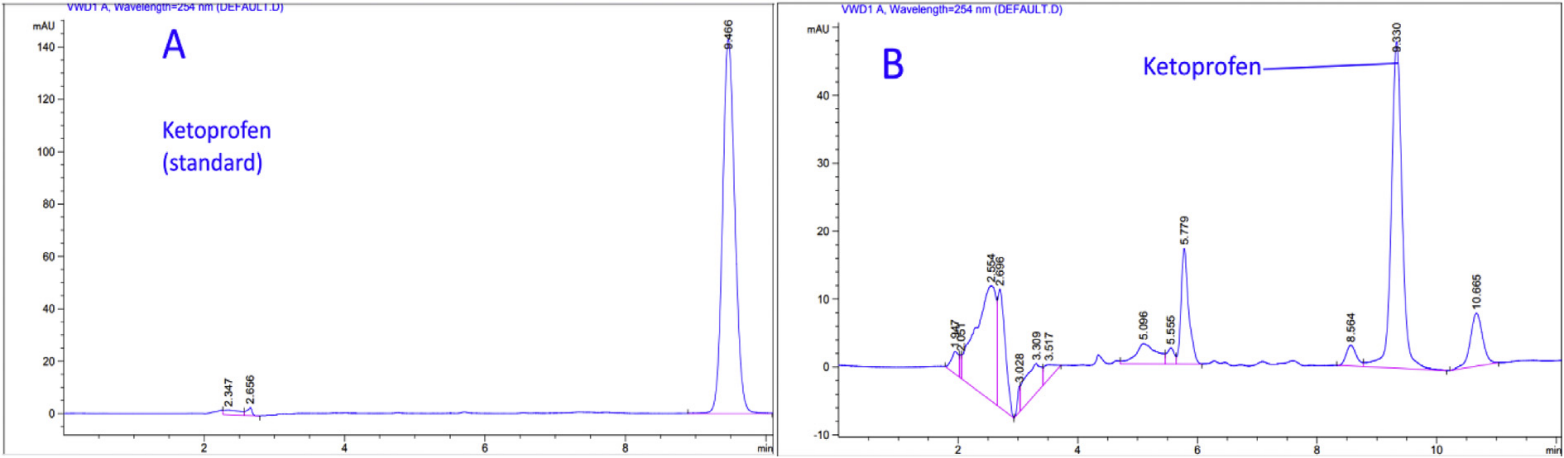
Chromatograms of (A) standard solution (0.250 mg/mL) and (B) Standing solution (0.250 mg/mL) of Ketoprofen.

**Figure 8:**
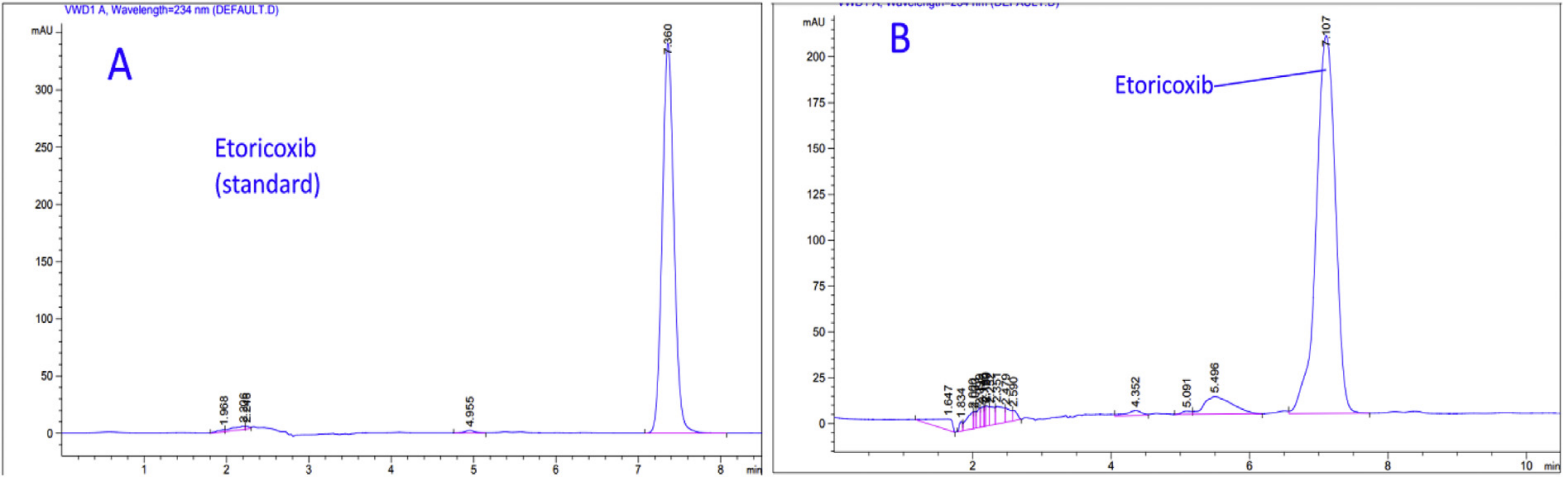
Chromatograms of (A) standard solution (0.058 mg/mL) and (B) Standing solution (0.058 mg/mL) of Etoricoxib.

**Figure 9:**
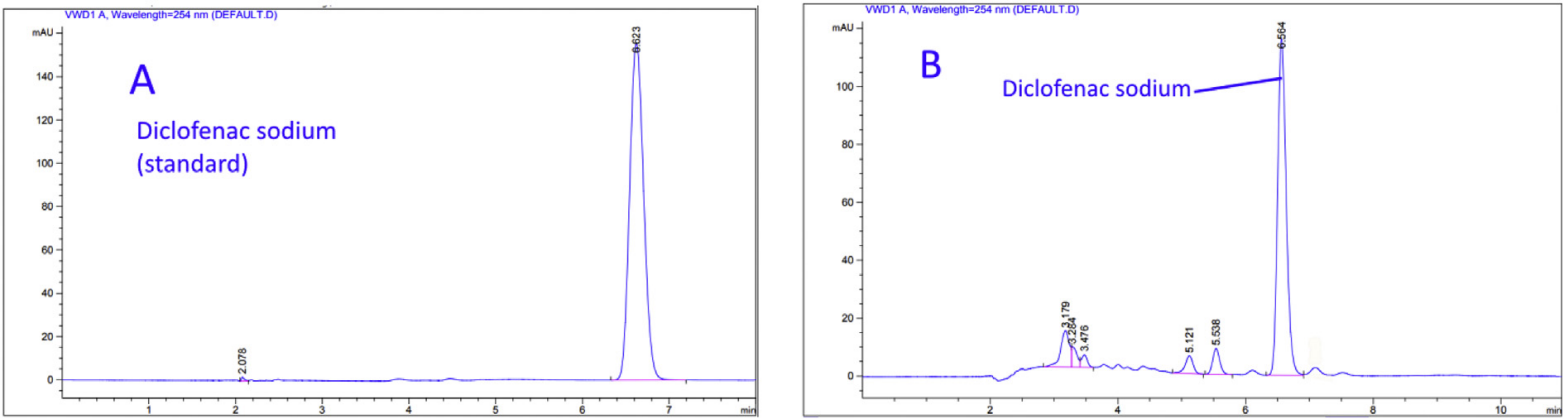
Chromatograms of (A) standard solution (0.125 mg/mL) and (B) Standing solution (0.125 mg/mL) of Diclofenac sodium.

In order to demonstrate the specificity of the proposed method, solutions of the three drugs were exposed to sunlight for 60 days at room temperature for degradation purposes, and then chromatograms of freshly prepared standard solutions and degraded ones were compared. Based on the resolution factor (Rs) of drug peak from the nearest resolving peak, it was noticed that degradation products were well resolved. Peaks of degradation substances have different retention times as opposed to drug peak. Concentrations of standard solutions used to investigate specificity were 0.250 mg/mL, 0.058 mg/mL, and 0.125 mg/mL for Ketoprofen, Etoricoxib, and Diclofenac sodium; respectively. Both chromatograms of standard solutions and the degraded ones are shown in (Figures 7, 8, and 9). Many additional peaks were well separated from the drug peak.

Regarding Ketoprofen, four additional peaks were before the main peak (${\text{R}}_{\text{t}}$= 2.5, 5.5, 5.7, and 8.5) and one peak after (${\text{R}}_{\text{t}}$= 10.6), where the resolution factor was (Rs = 5.03) (Figure 7). As for Etoricoxib, three additional peak appeared before the main peak and they were well resolved with a significant difference in the retention time (${\text{R}}_{\text{t}}$=1.8, 4.3, and 5.4), where the resolution factor was (Rs = 1.70) (Figure 8). For Diclofenac sodium, there were five additional peaks before the major peak of Diclofenac (${\text{R}}_{\text{t}}$= 3.1, 3.2, 3.4, 5.1, and 5.5). These peaks were well resolved with a significant difference in the retention time, where the resolution factor was (Rs = 9.50) (Figure 9). Ideal, (Rs) should be more than1. As a result, all (Rs) for the tested drugs were in the acceptance range indicating that the proposed method was significantly specific for the assay of Ketoprofen, Etoricoxib, and Diclofenac sodium in the presence of their degradation products.

#### 3.3.5 Ion-pair HPLC performance study

Three pharmaceuticals were selected for the study of ion-pair HPLC performance (Profenid®, Etoxia®, and Diclofenac Avenzor®). These pharmaceuticals were analyzed by two analysts in two different laboratories using two different HPLC instruments; (laboratory1: analyst1, Agilent 1260 infinity, Germany) and (laboratory2: analyst2, SHIMADZU CTO-20A, Japan). The optimal chromatographic conditions for the analysis of each pharmaceutical were applied to a different column than the one used in the validation study (C8 reverse-phase 150 × 4.6 mm, 5μm). Five different concentrations within the working range were chosen. Each concentration was injected three times. This process was repeated three different times within three weeks. The representative line of the relation between the areas under the curve (AUC) against the corresponding concentrations (C) for each pharmaceutical was constructed. Then, the correlation coefficients were calculated. The correlation coefficients were 0.9998, 0.9944, and 0.9999 for Profenid®, Etoxia®, and Diclofenac Avenzor®; respectively in laboratory1. While they were 0.9997, 0.9975, and 0.9980 for Profenid®, Etoxia®, and Diclofenac Avenzor®; respectively in laboratory2. Linear ranges were: (0.031–0.500 mg/mL) for Ketoprofen, (0.056–0.900mg/mL) for Etoricoxib, and (0.140–2.250 mg/mL) for Diclofenac sodium in both laboratories. Regression equations, correlation coefficients, and ranges were listed in Table 6. Results showed excellent linearity and correlations within the tested concentrations ranges using both HPLC instruments.

**Table 6:** Correlation coefficients, Regression equations, and ranges of profenid, profenid, and Diclofenac Avenzor.

		profenid®	Etoxia®	Diclofenac Avenzor®
Lab1^a^	Correlation coefficients (r)^C^	0.9998	0.9944	0.9999
	Regression equations^C^	y = 10858x + 83.59	y = 4477.20x + 183.17	y = 1053.10x + 6.96
	Ranges (mg/mL)	0.031–0.500	0.056–0.900 ​	0.140–2.250
Lab2^b^	Correlation coefficients (r)^C^	0.9997	0.9975	0.9980
	Regression equations^C^	y = 2E+07x + 80	y = 7E+06x + 38	y = 2E+06x - 40
	Ranges (mg/mL)	0.031–0.500	0.056–0.900 ​	0.140–2.250

a Agilent instrument was used to carry out HPLC analysis.

b SHIMADZU instrument was used to carry out HPLC analysis.

C n = 3: five concentrations over the working range.

Repeatability of the method was determined by injecting six replicate injections (on the same day) of each of the following concentration: 0.125 mg/mL, 0.225 mg/mL, and 0.562 mg/mL for Profenid®, Etoxia®, and Diclofenac Avenzor®; respectively and their corresponding responses were recorded. The mean of the areas was recorded with the least standard deviations. RSD% was calculated for each pharmaceutical in both laboratories. RSDs% were: 1.10%, 1.25%, and 0.15% for Profenid®, Etoxia®, and Diclofenac Avenzor®; respectively in laboratory1. While they were 0.91%, 1.80%, and 0.60% for Profenid®, Etoxia®, and Diclofenac Avenzor®; respectively in laboratory2. RSDs% obtained were less than 2% in both laboratories suggesting that the proposed method has excellent repeatability. Results were shown in Table 7.

**Table 7:** Results of repeatability study.

pharmaceuticals		AUC (mAU)						Mean^C^±SD	RSD%
		1	2	3	4	5	6		
Profenid® (0.125mg/mL)	Lab1^a^	1494.6	1460.0	1494.1	1463.1	1494.1	1488.5	1482.4 ± 16.3	1.10
	Lab2^b^	2089103.9	2092458.5	2092048.8	2075102.7	2127146.5	2085538.3	2095172 ± 19229.4	0.91
Etoxia® (0.225 mg/mL)	Lab1^a^	1296.5	1291.8	1251.5	1273.7	1274.3	1271.7	1276.6 ± 16.0	1.25
	Lab2^b^	2153253.7	2172070.7	2154821.7	2073253.3	2162722.7	2181048.4	2149528 ± 38821.5	1.80
Diclofenac Avenzor® (0.562 mg/mL)	Lab1^a^	604.9	603.1	603.4	604.9	605.4	605.0	604.5 ± 0.9	0.15
	Lab2^b^	1031831.5	1034817.2	1029255.0	1022165.0	1039774.8	1026421.0	1030710.7 ± 6226.1	0.60

a Agilent instrument was used to carry out HPLC analysis.

b SHIMADZU instrument was used to carry out HPLC analysis.

C n = 6.

**Figure 10:**
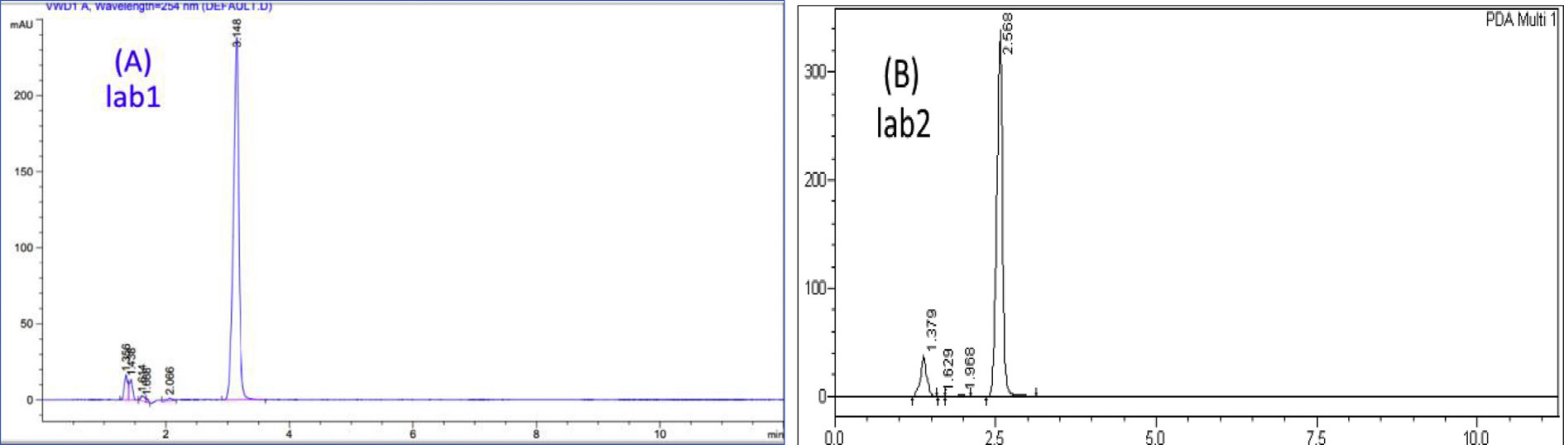
Chromatograms of profenid® (0.125 mg/mL) obtained (a) in laboratory1 by analyst1, using Agilent with a UV detector on C8 column; and (b) in laboratory2 by analyst2, using Shemadzu with a DAD detector on the C8 column.

**Figure 11:**
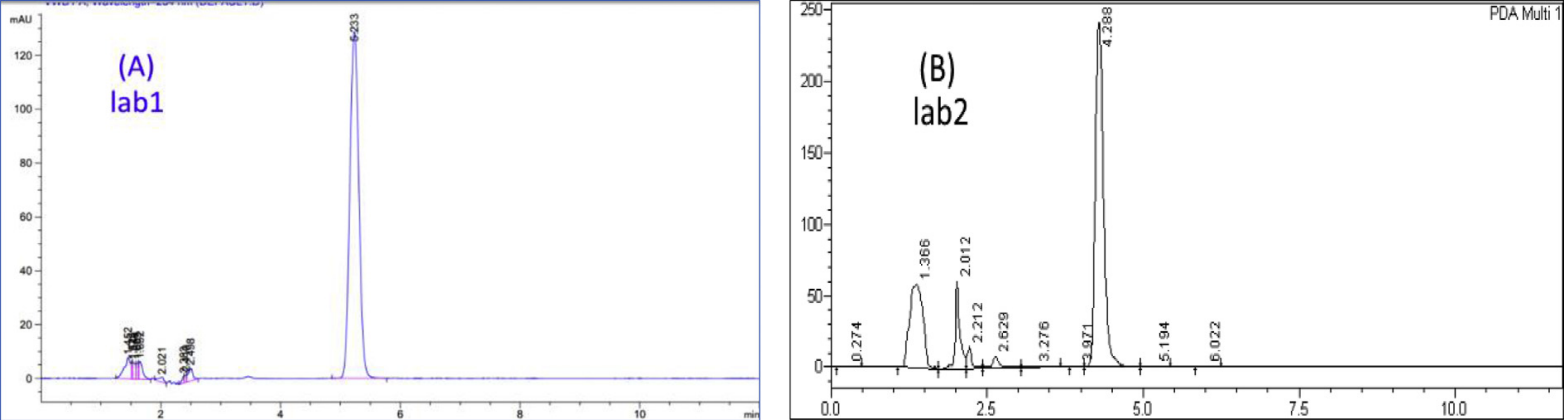
Chromatograms of Etoxia® (0.225 mg/mL) obtained (a) in laboratory1 by analyst1, using Agilent with a UV detector on C8 column; and (b) in laboratory2 by analyst2, using Shemadzu with a DAD detector on the C8 column.

**Figure 12:**
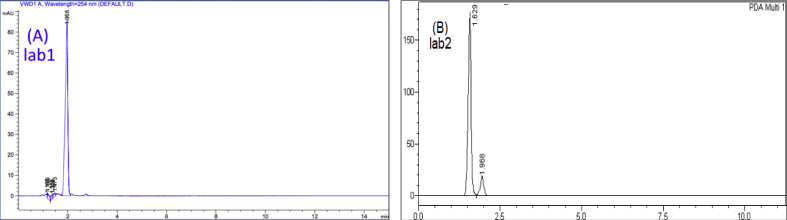
Chromatograms of Diclofenac Avenzor® (0.562 mg/mL) obtained (a) in laboratory1 by analyst1, using Agilent with a UV detector on the C8 column; and (b) in laboratory2 by analyst2, using Shemadzu with a DAD detector on the C8 column.

To evaluate the reproducibility, the measurement of 9 replicates of a previously fixed concentration for each pharmaceutical was repeated during a reasonable time (3 replicates in the same day, repeated three different times within three weeks). These procedures were performed taking into account several analytical variables (laboratory, HPLC instrument, analyst, and the prepared samples). The representative chromatograms for each pharmaceutical are presented in (Figures 10, 11, and 12). The mean of the areas and RSDs% were calculated. RSDs% were: 1.97%, 2.09%, and 0.98% for Profenid®, Etoxia®, and Diclofenac Avenzor®; respectively in laboratory1. While they were 0.80%, 1.60%, and 0.66% for Profenid®, Etoxia®, and Diclofenac Avenzor®; respectively in laboratory2. RSDs% obtained were less than 2% in both laboratories suggesting that the proposed method has a high reproducibility of results. Results were shown in Table 8.

**Table 8:** Results of reproducibility study.

pharmaceuticals		Profenid® (0.125 mg/mL)		Etoxia® (0.225 mg/mL)		Diclofenac Avenzor® (0.562 mg/mL)	
laboratory		Lab1^a^	Lab2^b^	Lab1^a^	Lab2^b^	Lab1^a^	Lab2^b^
AUC (mAU)	Week1	1494.6	2134317.6	1306.6	2157190	603.9	1031943
		1494.1	2099399.4	1293.9	2174254	604.1	1034928
		1494.8	2087515.5	1350.6	2171272	603.9	1029366
	Week2	1456.6	2089013.4	1271.8	2172823	605.4	1022085
		1561.3	2086529.3	1294.3	2192071	604.5	1039956
		1498.7	2118157.5	1270.7	2180048	605.0	1026532
	Week3	1465.1	2093058.8	1271.2	2074253	615.0	1023916
		1480.02	2085102.7	1270.1	2174254	615.6	1019680
		1491.6	2093458.5	1270.2	2174822	618.2	1021587
Mean^C^ ±SD		1493.0 ± 29.5	2098505.8 ± 16802.4	1288.8 ± 26.9	2163443 ± 34644.0	608.4 ± 5.9	1027777 ± 6819.7
RSD%		1.97	0.80	2.09	1.60	0.98	0.66

a Agilent instrument was used to carry out HPLC analysis.

b SHIMADZU instrument was used to carry out HPLC analysis.

C n = 9.

### 3.4 Pharmaceuticals assay

One Pharmaceutical was analyzed for Ketoprofen and two Pharmaceuticals were analyzed for both Etoricoxib and Diclofenac sodium using the described method. Quantification was carried out in triplicates and the obtained results were presented in Table 9.

**Table 9:** Results of pharmaceuticals assay using the developed HPLC method.

pharmaceuticals	profenid®	Toricox®	Etoxia®	Diclorism®	Diclofenac Avenzor®
Active ingredient and potency	ketoprofen 50mg	Etoricoxib 60mg	Etoricoxib 90mg	Diclofenac sodium 50mg	Diclofenac sodium 75mg
Pharmaceutical form	capsule	tablet	tablet	tablet	ampoule
Manufacturer name	Oubri	Unipharma	Razi	Shifa	Avevzor
Country of production	(Syria)	(Syria)	(Syria)	(Syria)	(Syria)
Units Number	20	20	20	20	5
Area Under The Curve (AUC)	1393.0	1349	1254	633.4	956.9
	1394.0	1351	1257	633.5	951.4
	1390.8	1345	1261	633.3	949.6
Found values (mg/mL)	0.197	0.024	0.022	0.047	0.072
	0.197	0.024	0.022	0.047	0.071
	0.196	0.024	0.022	0.047	0.071
Mean^a^±SD	0.197 ± 0.02	0.024 ± 0.005	0.022 ± 0.006	0.047 ± 0.0006	0.072 ± 0.02
RSD %	0.12	0.21	0.26	0.01	0.39
Theoretical concentrations^b^ (mg/mL)	0.200	0.024	0.024	0.050	0.074
content %	98.57	101.54	94.96	95.46	96.00

a n = 3.

b appropriate concentrations in the range of linearity, which they were prepared by diluting an amount equivalent to the labeled content of each pharmaceutical. (Mentioned in samples solutions preparation paragraph).

The acceptable range mentioned in USP Pharmacopeia for Ketoprofen capsules is 90%–110% of the labeled amount [48]. The actual content of Ketoprofen in Profenid® was 98.57% of the labeled claims with RSD% = 0.12. Although there isn't a monograph in the USP for Etoricoxib tablet however, the allowable range for other NSAIDs is mostly 95% and 105%. The actual contents of Etoricoxib in Toricox® and Etoxia® were 101.54% and 94.96%; respectively of the labeled claims with RSD%= (0.22 and 0.27). The acceptable range mentioned in USP Pharmacopeia for Diclofenac tablet is 90%–110% of the labeled amount. The actual content of Diclofenac in Diclorism was 95.46% of the labeled claims with RSD% = 0.01. Although there isn't a monograph in the USP for Diclofenac ampoules however, the allowable range for other NSAIDs is mostly 95% and 105%. The actual content of Diclofenac in Diclofenac Avenzor® was 96.00% of the labeled claims with RSD% = 0.39. All the above results were in accordance with the official requirements. The content of Ketoprofen capsules was determined by Yen SU et al [49]; the value was 101% as the percentage of the labeled claim. Etoricoxib was determined in their formulations by Krishna R Gupta et al [50] and the value was 99.90%. Diclofenac tablets were assayed by Sunil R. DH, Vidhya K. Bh [51] and the result was 99.93% of the labeled claim.

### 3.5 Comparative study among developed and announced HPLC approaches

Many previous chromatographic methods were used to analyze NSAIDs using HPLC. Depending on the comparative study between the developed and the announced HPLC approaches, disadvantages like precipitations and blockages in the chromatography column during the analysis process were common with previous classical methods because of using buffers in the mobile phases. However, using Cetrimide as a surfactant in mobile phase instead of buffers helps the developed method overcome those drawbacks. Reduction of salts’ precipitation in the pump, improvement of the flow ability of the mobile phase, and decreasing pressure applied to the column during the analysis process was noticeable in the developed method. Moreover, some pharmaceutical compounds which were analyzed by this method showed a shorter retention time as opposed to classical methods. Besides, the use of minimal organic solvent made this method an environmental friendly technique. On the other hand, this method still suffers challenges such as limitations to analyze cationic charged ionisable compounds. Table 10 shows a comparison of the present work performance with previously published works. Our employed approach shows a better result in terms of several performance measures. Regarding Ketoprofen, the proposed method has more precise results than the method of Y. Sun et al [49]. M.J. Martı'n [52] used aqueous formic acid/formate buffer and methanol as a mobile phase in the ratio 90:10. Ketoprofen was separated with (${\text{R}}_{\text{t}}$=14). While the buffer was replaced by ion-pair reagent and the analysis was applied with less retention time (${\text{R}}_{\text{t}}$=9.4) using 50% acetonitrile: 50% water in our approach. As for Etoricoxib, the analysis was applied with least retention time (${\text{R}}_{\text{t}}$=7.3) without using any buffers in the mobile phase. The method used by K.R. Gupta et al [50] showed better specificity (Rs = 5%) using a mobile phase consisted of acetonitrile, methanol, and water in the ratio 60:15:25 than our method. For Diclofenac sodium, the method used by D.J. Bhat [55] showed better retention time (${\text{R}}_{\text{t}}$=2.9) with recovery% value equal to 100.73% using a mobile phase consisted of Methanol: Water in the ratio 90:10 comparing to (${\text{R}}_{\text{t}}$=6.66) using a mobile phase consisted of acetonitrile: Water in the ratio 70:30 in the proposed method. It should be mentioned that this study is the first to provide a valid, single, and ion-pair liquid chromatography method to determine three drugs at the same time, Ketoprofen, Etoricoxib, and Diclofenac sodium in bulk and pharmaceuticals. All previous advantages of the proposed approaches beside fewer drawbacks compared to the announced ones as presented in Table 11 give them the priority of application in the quality control laboratory.

**Table 10:** Comparative study between some previously published HPLC studies and the presented study.

	Column (mm)	Mobile phase constituents	Detection wavelengths (nm)	performance						Reference
				Linearity (r)	Range	Accuracy (Recovery %)	Precision (RSD %)	Specificity (Rs)	Retention times (R_t_) (min)	
**Ketoprofen**	ODS 250 × 4.6	Acetonitrile: acetat buffer: methanol: (35:40:25)	255	0.9999	0.1–100 μg/mL	97.10	5.0	–	9	[49]
	ODS2 150 × 4.6	Aqueous formic acid/formate buffer (0.1 M): methanol (90:10)	254	0.9990	5–35 mg/L	98.20	Less than 1	–	14.3	[52]
	**C18** **250 × 4.6**	**ACN: water with cetrimide**$\;10^{-3}$**M (50:50)**	**254**	**0.9999**	**0.03- 0.50 mg/mL**	**98.90**	**0.41**	**5.03**	**9.4**	**presented study**
**Etoricoxib**	C18 250 × 4.6	Acetonitrile: methanol: water (60:15:25)	236	0.9996	1–5 μg/mL	99.66	less than 2%	5.07	7.6	[50]
	C18 250 × 4.6	acetonitrile: methanol: KH2PO4 buffer	234	0.9998	25–400 ng/20μL	99.83± 0.55	0.28–1.36%	–	8	[53]
	**C18** **250 × 4.6**	**ACN: water with cetrimide**$\;10^{-3}$**M (50:50)**	**234**	**0.9998**	**0.007-0.11 mg/mL**	**99.45**	**0.14**	**1.7**	**7.3**	**presented study**
**Diclofenac sodium**	C18 250 × 4.6	ACN:Phosphatebuffer (50:50)	220	0.9980	6–16 μg/mL	98.55	0.013	–	9.1	[54]
	C18 250 × 4.6	Methanol: Water (90:10)	269	0.9990	10–60 μg/mL	100.73± 0.38	1.30	–	2.9	[55]
	**C18** **250 × 4.6**	**ACN: water with cetrimide**$\;10^{-3}$**M (70:30)**	**254**	**0.9999**	**0.016 - 0.250 mg/mL**	**99.49**	**0.09**	**9.5**	**6.6**	**presented study**

**Table 11:** Comparative study between the previous published HPLC study and the presented study.

Method	Advantages	Drawbacks
HPLC- NP	✓The use of Non-polar solvent to dissolve a sample. ✓Perfect for isomer isolation, very hydrophilic or hydrophobic molecules. ✓Low viscosity solvents, higher flow rates. ✓Can be used for compounds that can decompose in water.	• Is not suitable for the analysis of a wide range of compounds. • Gradient elution is not possible. • Retention time of components can be variable. • The control of the solvent strength is difficult. • Requires adding buffers in the mobile phase.
HPLC- RF	✓The possibility of using water in the mobile phase with other solvents. ✓Accurate results with small amounts of sample. ✓The pH selectivity can be used to improve the separation. ✓The hydrophobic stationary is suitable for the retention of organic molecules.	• Increased difficulty to analyze Water-insoluble compounds. • The silica of the reverse-phase column can be dissolution at pH > ∼7.5. • Eluted sample cannot be recovered. • Requires adding buffers in the mobile phase.
HPTLC	✓Low cost, rapid, doesn't need expensive equipment.	• Low range of repeatability, low level of automation.
HPLC/UHPLC	✓Versatile ✓Rapid, automated, doesn't require large samples, delivers high repeatability.	• The sample has to be highly purified, high equipment and depreciation coast. • Requires adding buffers in the mobile phase.
HPLC –IP (presented study)	✓Improvement of the flow ability of the mobile phase. ✓The decreasing of applied pressure on the column. ✓Improvement of peak shapes. ✓Reduces separation time for some compounds. ✓The broadest selection of carbon chain length for better separation and retention. ✓Minimal organic solvent, environmental friendly technique. ✓No requirement for the adding of buffers in mobile phase. ✓Precise and reproducible results.	• Column equilibration takes a longer time after changing the mobile phase. • The coast of analysis is a little high.

### 3.6 Postulated mechanism for the ion-pair reagents on C18 column

Ion-Pair liquid chromatography technique has gained a wide acceptance to separate ionic solutes, unlike other methods like ion exchange and ion suppression. They are limited to separate neutral compounds and suffer difficulties in separating ionic components by the reverse-phase. Therefore, the ion-Pair liquid chromatography technique was selected in this study especially that NSAIDs have ionsable chemical structure [1].

**Figure 13:**
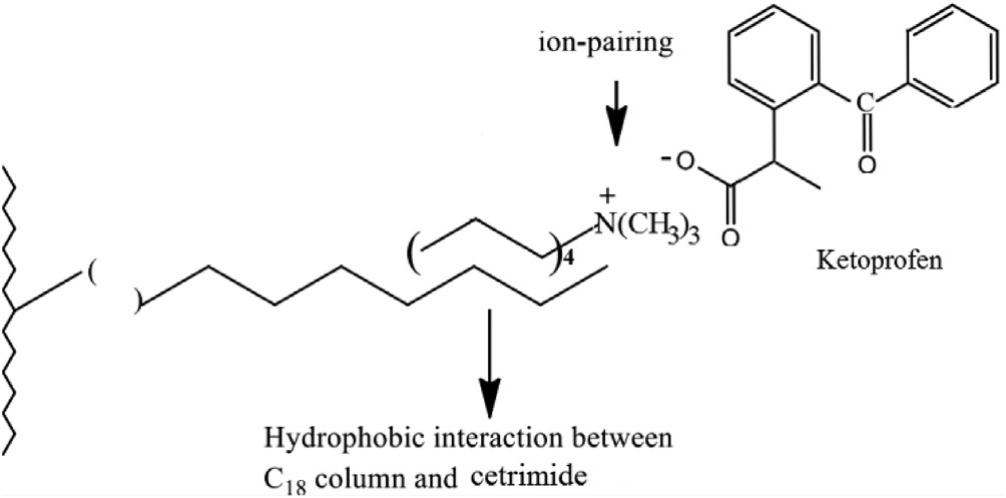
Proposed ion–pair mechanism of Cetrimide with Ketoprofen on C18 column.

**Figure 14:**
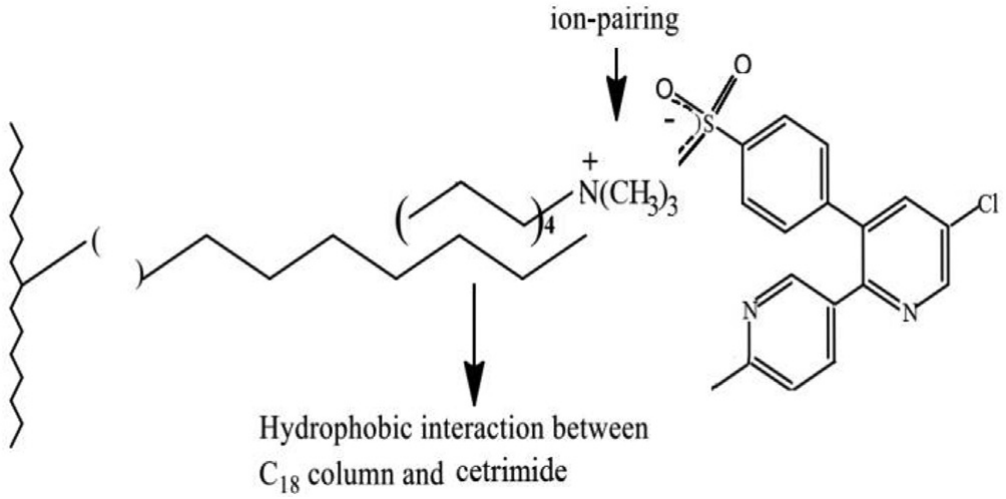
Proposed ion–pair mechanism of Cetrimide with Etoricoxib on C18 column.

**Figure 15:**
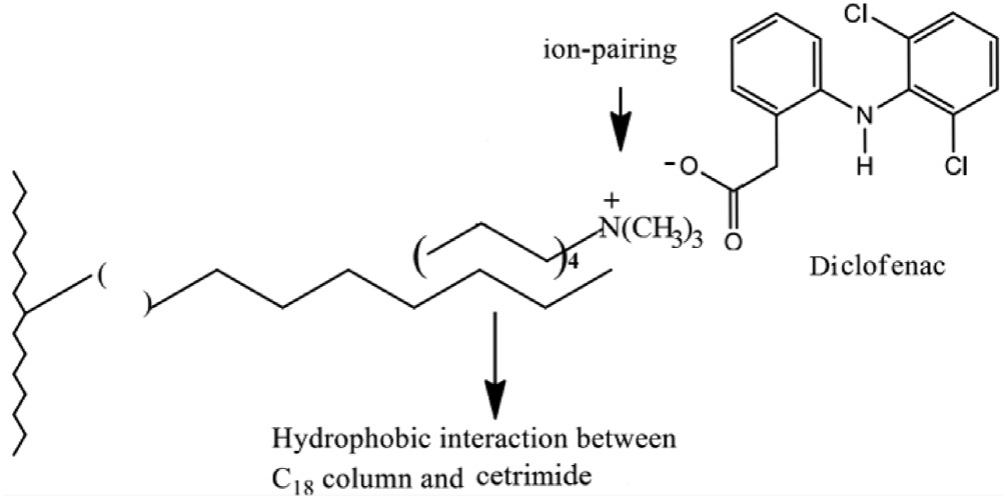
Proposed ion–pair mechanism of Cetrimide with Diclofenac on C18 column.

The postulated mechanism for the ion-pair complex of test drugs is illustrated in (Figure 13) (Figure 14), and (Figure 15). The pH of the mobile phase is adjusted to10 using ammonium hydroxide. Therefore, it is reasonable to assume that the addition of weak organic acids to a basic medium (pKa ˂ pH) will facilitate the deprotonation of analytes in a negative-ion mode such as (–${\text{coo}}^{-}$) of (Ketoprofen and Diclofenac) and (–${\text{soo}}^{-}$) of Etoricoxib [56].

The ion-pair complex is formed between the positive charge of trimethylammonium ion of Cetrimide [${\text{N}\left({\text{CH}}_{3}\right)}_{3}^{+}$] and the negative charge of carboxylate ion (–${\text{coo}}^{-}$) of (ketoprofen and Diclofenac) or the negative charge of sulfonyl ion (–${\text{soo}}^{-}$) of Etoricoxib. The carbon chain in Cetrimide undergoes hydrophobic alignment with the C18 chain which is responsible for moderate retention of Ketoprofen, Diclofenac, and Etoricoxib in stationary phase.

## 4 Conclusion

Various NSIDs (Ketoprofen, Etoricoxib, and Diclofenac sodium) were determined in pure and pharmaceuticals using a single, simple, and novel ion-pair HPLC method.

This method was developed and validated with high selectivity and very little use of organic solvent. The use of a minimal organic solvent in HPLC made this method an environmental friendly technique. The addition of a surfactant (Cetrimide) to the mobile phase instead of buffers improved the flow ability of the mobile phase, decreased salts' participation, reduced analysis time, and improved the peaks’ shape. Because of those advantages and applicability for the quantitative and qualitative analyzing of Ketoprofen, Etoricoxib, and Diclofenac sodium in dosage forms, this method is considered valuable and practical for the routine application of the assay of Ketoprofen, Etoricoxib, and Diclofenac sodium in quality control laboratories.

## Declarations

### Author contribution statement

G.M. Andraws: Performed the experiments; Analyzed and interpreted the data; Contributed reagents, materials, analysis tools or data; Wrote the paper.

S. Trefi: Conceived and designed the experiments.

### Funding statement

This research did not receive any specific grant from funding agencies in the public, commercial, or not-for-profit sectors.

### Competing interest statement

The authors declare no conflict of interest.

### Additional information

No additional information is available for this paper.
